# Editorial: Epigenomic contributions to autism spectrum disorders

**DOI:** 10.3389/fnins.2023.1177378

**Published:** 2023-04-18

**Authors:** Starnawska Anna, Janecka Magdalena

**Affiliations:** ^1^Department of Biomedicine, Aarhus University, Aarhus, Denmark; ^2^The Lundbeck Foundation Initiative for Integrative Psychiatric Research, iPSYCH, Aarhus, Denmark; ^3^Center for Genomics and Personalized Medicine, CGPM, and Center for Integrative Sequencing, iSEQ, Aarhus, Denmark; ^4^Seaver Autism Center, Icahn School of Medicine at Mount Sinai, New York, NY, United States; ^5^Department of Psychiatry, Icahn School of Medicine at Mount Sinai, New York, NY, United States; ^6^Department of Genetics and Genomic Sciences, Icahn School of Medicine at Mount Sinai, New York, NY, United States

**Keywords:** epigenetics, DNA methylation, environmental risk, autism, psychiatry

## Multifactorial origins of autism spectrum disorder

Autism spectrum disorder (ASD) is a complex neurodevelopmental disorder characterized by deficits in social communication along with repetitive and restrictive behaviors. ASD affects 1%−1.5% of individuals in the general population, and is diagnosed ~3–4.5 times more commonly in males than females (Developmental Disabilities Monitoring Network Surveillance Year 2010 Principal Investigators; CDC, 2014; Loomes et al., 2017). It is a highly heritable disorder with twin-heritability 64%−91% (Tick et al., 2016) and SNP-heritability reaching 0.12 (Grove et al., 2019).

Apart from the common genetic variation, rare *de novo* mutations, such as gene-disrupting point mutations and copy number variants, contribute substantially to the individual ASD liability, both as standalone risk factors, and jointly with the polygenic burden (Gaugler et al., 2014; Iossifov et al., 2014; Grove et al., 2019; Klei et al., 2021).

In addition to the genetic factors, population- and family-based studies reported environmental factors to also affect liability to ASD (Hallmayer et al., 2011). Increasing evidence suggests the potential role of multiple environmental factors in development of ASD, including perinatal exposures like environmental and chemical pollutants (organophosphates, phthalates), short interpregnancy interval, advanced paternal age, lifestyle and diet (maternal folic acid, prenatal vitamins, and iron levels), maternal infections during pregnancy, pre-eclampsia, and gestational diabetes, among others (Schmidt et al., 2012, 2014; Shelton et al., 2014; Walker et al., 2015; Conde-Agudelo et al., 2016; Nahum Sacks et al., 2016; Janecka et al., 2017; Hertz-Picciotto et al., 2018; Hornig et al., 2018). While the mechanisms underlying these associations remain not fully understood, epigenomic regulation of the genome is considered to be one of the molecular mechanisms that allows both genetic and environmental risk factors to modulate phenotypic variance (Smith et al., 2014; Yet et al., 2016; Martin and Fry, 2018).

## Epigenetic factors in ASD

Epigenetics, literally meaning “above the genetics,” refers to heritable changes in gene regulation and expression that do not involve changes to the underlying DNA sequence. The most widely studied epigenetic regulators of gene expression, genome regulation, and genome stability are DNA methylation, histone modifications, and nucleosome positioning (Birney et al., 2007; Gershman et al., 2022), with majority of current research focused on investigating associations between the levels of DNA methylation across the genome and a phenotype of interest. Multiple lines of evidence support the notion of epigenetic dysregulation in ASD, including genes encoding epigenetic machinery being strongly implicated in the disorder [e.g., genetic variation in chromatin modifiers (Cukier et al., 2010; Abrahams et al., 2013)], and abnormally methylated loci across the genome identified in ASD cases (Tremblay and Jiang, 2019). Moreover, the recently identified ASD risk SNPs physically interact, through chromatin looping, with genes crucial for fetal brain development, expression of which is dynamically changed and regulated during fetal corticogenesis (Grove et al., 2019). With accumulating evidence on the role of epigenetic regulation in molecular pathophysiology of ASD—a neurodevelopmental disorder, with likely perinatal origins—there has been an increase in epigenetic association studies of the disorder across diverse, disorder-relevant tissues (umbilical cord blood, placenta, blood, sperm, and various brain regions)—encouraging further epigenomic research of this complex disorder (Andrews et al., 2018; Kimura et al., 2019; Zhu et al., 2019; Mordaunt et al., 2020; García-Ortiz et al., 2021; Garrido et al., 2021).

## Research Topic overview

This Research Topic aimed to gather interdisciplinary contributions to widen our knowledge on the epigenetic dysregulation due to downstream effects of ASD genetic and environmental risk factors. The issue includes six articles (Lei et al.; Wang et al.; Mouat and LaSalle; Santos et al.; Urbonaite et al.; Takahashi et al.), three primary research papers, and three literature reviews. The epigenetic enquiry in the articles covers research in alterations to DNA methylation patterns, expression of enzymes responsible for methylation and demethylation processes of DNA (DNMTs and TET2) or enzymes modifying histone code (HDAC). The main focus of the articles was to investigate impact of prenatal environmental ASD risk factors (such as maternal high fat diet, hypoxia, stress) on epigenetic changes in offspring, with potential consequences for neurodevelopment and neuropsychiatric disease risk. In addition to investigating environmental exposures, one of the articles explored the gene-environment interaction hypothesis of origins of ASD. In the study, authors suggested that increased ASD risk associated with xenobiotic exposure may be due to increased vulnerability to these compounds in carriers of damaging variants in genes responsible for xenobiotic detoxification, and that such combination of genetic susceptibility and environmental insult could potentially result in ASD (Santos et al.). Lastly, one of the articles included in this Research Topic is a case report that investigated a potential link between epigenetic regulation and structural changes in ASD postmortem brain tissues (Takahashi et al.).

## Converging findings from diverse approaches

Articles included in this thematic issue utilized different research approaches (original research, literature review) and molecular techniques (DNA methylation quantification at candidate loci by pyrosequencing or at whole-genome level by bisulfite sequencing, measurements of expression of epigenome regulators) to investigate whether, and how, regulation of the genome can contribute to development of ASD. Overall, the studies provide support that potential environmental risk factors of ASD, such as maternal high fat diet, prenatal exposure to stress, or birth hypoxia, may alter the epigenome—thus suggesting putative molecular mechanism which can mediate effects of these environmental exposures.

The literature review by Urbonaite et al. on molecular changes associated with maternal high fat diet highlights sexually dimorphic changes in global DNA methylation patterns associated with the exposure. These changes occurred across various brain regions, and included both hypo- and hyper-methylation depending on the investigated tissue (Urbonaite et al.). Maternal high fat diet was also reported to have a stronger effect (dysregulated expression of higher number of genes) during embryonic brain development in males, compared to females (Edlow et al., 2016), which the authors proposed could be due to sexual dimorphic changes in DNA methylation associated with the exposure (Urbonaite et al.). A similar phenomenon was reported in another study included in this thematic issue by Lei et al., who reported that in rodents prenatal stress was associated with sex-specific changes of expression of enzymes responsible for genome methylation and demethylation processes in the brain tissue. The phenotypic consequences of prenatal stress on offspring were also sex-specific, with anxiety-like behavior observed especially in females and depression-like behavior in males (Lei et al.). It is well-established that the trajectories of DNA methylation changes in the embryonic brain tissue differ between males and females (Spiers et al., 2015), and that these dynamic changes are accompanied by fluctuations of expression of methylome-modifying proteins DNMTs and TETs in brain tissue during this period (Miller et al., 2014). Therefore, with the additional evidence suggesting that environmental prenatal risk factors for ASD may alter brain DNA methylation levels in a sex-specific manner, it is conceivable to hypothesize that the epigenetic regulation may contribute to the differences in ASD prevalence between males and females (Developmental Disabilities Monitoring Network Surveillance Year 2010 Principal Investigators; CDC, 2014; Loomes et al., 2017).

Moreover, this Research Topic includes an extensive literature review highlighting that ASD environmental risk factors may be associated with epigenetic changes (investigated most extensively in the context of DNA methylation) not only in the exposed generation, but also inter-generationally (Mouat and LaSalle). These observations could suggest putative molecular mechanism for an increased rates of ASD and ASD-related phenotypes in several generations following an environmental insult (Mouat and LaSalle). Nevertheless, mechanistic understanding of how variable DNA methylation levels impact human brain development and ASD risk, and how these effects may persist transgenerationally in humans, will require a new set of carefully designed studies.

The majority of the epigenomic research presented in this thematic issue was conducted, or reviewed in animals, and therefore, their findings still need to be replicated in human subjects. The only human case-report original research epigenetic study included in this thematic issue quantified and compared genome-wide DNA methylation levels in healthy vs. ASD brain tissue (Takahashi et al.). The study findings suggest epigenetic dysregulation in genes related to neuronal development to be associated with the disorder. This is in line with evidence from the previous studies on changes in brain DNA methylation in several brain regions of ASD cases (such as Brodmann area, dorsolateral prefrontal cortex, temporal cortex, cerebellum) (Ladd-Acosta et al., 2014; Nardone et al., 2014; Ellis et al., 2017). Additionally, one of the largest epigenetic ASD studies performed in the brain tissue (prefrontal cortex, temporal cortex, cerebellum) reported multiple changes in DNA methylation levels to be associated with ASD, and ASD-related co-methylation modules to be significantly enriched for synaptic, neuronal, and immune dysfunction genes (Wong et al., 2019). These findings are still pending replication in larger cohorts, to ensure sufficient statistical power for these discoveries—which, due to limited access to human brain tissue, has been one of the greatest challenges in molecular research of neuropsychiatric disorders.

In addition to changes in epigenetic regulation upon exposure to environmental risk factors of ASD, previous studies demonstrated that genetic risk variants for mental disorders are associated with variation in DNA methylation (Starnawska and Demontis, 2021). This phenomenon was also reported for ASD genetic risk variants, which were not only shown to act as methylation quantitative trait loci (mQTLs), but an additive (polygenic) burden of ASD was also associated with differential blood DNA methylation patterns already at birth (Hannon et al., 2018). The articles included in this thematic issue did not include data on impact of ASD genetic risk variants on epigenetic regulation, which at the time of publishing this Research Topic is not uncommon in epigenetic research of ASD. Therefore, this thematic issue not only collects articles on epigenomic contributions to ASD and ASD-related phenotypes, but also encourages combining environmental, genetic and epigenetic data in the future research of ASD to provide deeper understanding of its molecular pathophysiology.

## Perspectives

ASD is a complex and multifactorial neurodevelopmental disorder which risk is modulated by multiple genetic, environmental and epigenetic risk factors (Havdahl et al., 2021; Mathew et al., 2021). This Research Topic collected multidisciplinary investigations of associations between changes in epigenetic regulation of the genome and environmental ASD risk factors, as well as ASD diagnosis itself. Studies included in this thematic issue expand our knowledge on how dysregulation of epigenetic regulation could modulate ASD risk. Nevertheless, in order to gain deeper understanding of epigenetic contributions to ASD many challenges remain, and carefully designed studies are still needed to fill the abovementioned knowledge gaps. Future epigenomic studies of ASD should therefore focus on:

1) Creating large biobanks of human brain tissue and utilizing them for analysis of epigenetic dysregulation associated with ASD across development. Collection of such large cohorts will help ensure sufficient statistical power for the association analyses, and may therefore (i) enable testing replication of the existing findings and (ii) lead to discovery of novel epigenetic contributions to the molecular pathophysiology of the disorder.
2) Investigating in parallel many types of epigenetic modifications from the same tissue. Currently, most studies still investigate associations between the phenotype of interest and one epigenetic modification at a time. However, the epigenomic regulation of the genome and gene expression is a well-orchestrated process, with multiple epigenetic modifications acting in tandems to regulate chromatin accessibility. Therefore studying e.g. DNA methylation and histone modifications together will allow for detailed mapping of the dysregulation of the epigenetic landscape in complex neuropsychiatric disorders.
3) Collecting multi-generational environmental, genetic, epigenetic and epidemiological data and using them jointly in neuropsychiatric research, in order to gain deeper understanding on changes in ASD risk across multiple generations following an environmental insult. Additionally, such datasets can be a valuable resource for studying contributions of gene-environment interactions to development of ASD.
4) Applying diverse statistical approaches, such as Mendelian randomization, to study causal effects of environmental exposures, genetic risk variants, and epigenetic regulation on ASD. Use of such methods will allow to disentangle to what extend environmental and genetic risk factors of ASD act through epigenetic factors to cause the disorder.

## Author contributions

All authors listed have made a substantial, direct, and intellectual contribution to the work and approved it for publication.
